# 

**Published:** 1905-10

**Authors:** 


### CONTENTS.

ORIGINAL COMMUNICATIONS—

AUTOINTOXICATION. By D. L. Field, M.D. .... 433
WHAT I KNOW ABOUT HAY FEVER. By E. Van Goidtsnoven, M.D., Atlanta, Ga. .... 443
ANKYLOBLEPHARON. By Wm. Lewis Bullard, M.D., Columbus, Ga. .... 451
REPORT OF A CASE OF TYPHOID FEVER WITH UNUSUAL FEATURES. By G. Pope Huguley, M.D., Atlanta, Ga. .... 452
ETIOLOGY AND PREVENTION OF PERINEAL LACERATIONS, WITH SPECIAL REFERENCE TO THE SHOULDERS. By Archibald Smith, M.D., Atlanta, Ga. .... 455
DOGMAS AND FASHIONS IN PRACTICE. By E. C. Cartledge, M.D., Atlanta, Ga. .... 463

EDITORIAL NOTES AND COMMENTS—

HEALTH BOARDS AND QUARANTINES .... 471

NEWS AND NOTES .... 474
CONTENTS CONTINUED .... X

